# Host cell interactions of novel antigenic membrane proteins of *Mycoplasma agalactiae*

**DOI:** 10.1186/s12866-022-02512-2

**Published:** 2022-04-08

**Authors:** Maysa Santos Barbosa, Lucas Miranda Marques, Jorge Timenetsky, Renate Rosengarten, Joachim Spergser, Rohini Chopra-Dewasthaly

**Affiliations:** 1 Institute of Microbiology, Department of Pathobiology, University of Veterinary Medicine Vienna, Veterinaerplatz 1, Vienna, A-1210 Austria; 2 Present Address: Department of Microbiology, Institute of Biomedical Science, University of São Paulo, São Paulo, Brazil; 3 Multidisciplinary Institute of Health, Federal University of Bahia, Vitória da Conquista, Brazil

**Keywords:** Contagious agalactia, Cell adhesion, Cytopathic effects, ECM interaction, Plasminogen binding proteins, Immunomodulation, Mycoplasma pathogenicity

## Abstract

**Background:**

*Mycoplasma agalactiae* is the main etiological agent of Contagious Agalactia syndrome of small ruminants notifiable to the World Organization for Animal Health. Despite serious economic losses, successful vaccines are unavailable, largely because its colonization and invasion factors are not well understood. This study evaluates the role of two recently identified antigenic proteins (MAG_1560, MAG_6130) and the cytadhesin P40 in pathogenicity related phenotypes.

**Results:**

Adhesion to HeLa and sheep primary mammary stromal cells (MSC) was evaluated using ELISA, as well as in vitro adhesion assays on monolayer cell cultures. The results demonstrated MAG_6130 as a novel adhesin of *M. agalactiae* whose capacity to adhere to eukaryotic cells was significantly reduced by specific antiserum. Additionally, these proteins exhibited significant binding to plasminogen and extracellular matrix (ECM) proteins like lactoferrin, fibrinogen and fibronectin, a feature that could potentially support the pathogen in host colonization, tissue migration and immune evasion. Furthermore, these proteins played a detrimental role on the host cell proliferation and viability and were observed to activate pro-apoptotic genes indicating their involvement in cell death when eukaryotic cells were infected with *M. agalactiae*.

**Conclusions:**

To summarize, the hypothetical protein corresponding to MAG_6130 has not only been assigned novel adhesion functions but together with P40 it is demonstrated for the first time to bind to lactoferrin and ECM proteins thereby playing important roles in host colonization and pathogenicity.

**Supplementary Information:**

The online version contains supplementary material available at 10.1186/s12866-022-02512-2.

## Introduction

Although mycoplasmas have a reduced genome they can behave as complex microorganisms [1, 2]. In the absence of a cell wall, important interactions with the host cells are carried out by their cytoplasmic membranes [3–5]. In mycoplasmas, the integral and membrane-associated proteins are exposed to the environment and play an important role in the survival and pathogenesis of the agent [1, 5].

Mycoplasmas have several lipid-associated membrane proteins (LPPs) which are able to modulate immune responses [5, 6]. Some important LPPs in *Mycoplasma* spp. have been described, such as LppQ in *M. mycoides* subsp. *mycoides* [7]; P60 in *M. capricolum* subsp. *capricolum* [8] and P30 in *M. pneumoniae* [9]. Mycoplasma LPPs are important virulence factors and targets of growth inhibitory antibodies, and may influence several functions such as apoptosis [10], antigenic variation [11], transport of molecules [12], nuclease activity [13] and adhesion [14, 15].

In case of *M. agalactiae,* the main causative agent of Contagious agalactia syndrome in sheep and goats, few membrane proteins have been identified, such as the P30 protein [16], cytadhesin P40 [17], P48 protein [18, 19], lipoprotein MAG_5040 [13], pyruvate dehydrogenase [20] and Vpmas [21, 22]. In addition, MAG_1560 and MAG_6130 were identified by our group as novel antigenic proteins using bioinformatic analyses and demonstrated reactivity in immunoassays to sera from infected goats/sheep sera [23]. Since MAG_1560 and MAG_6130 were identified as membrane immunogenic proteins together with P40, a known cytadhesin of *M. agalactiae* [23], this study aimed to elucidate their functions. Adherence being a fundamental step for microbial colonization and infection [24, 25], these two hypothetical proteins were also evaluated for their role in adhesion to host cells together with P40 cytadhesin. Protein-protein interactions involving adhesins and components of the host extracellular matrix are integral and recurring features of bacterial pathogens [25] and were also analyzed. As bacterial adhesion is known to alter cell signaling to facilitate the spread of the pathogens by host immune evasion, internalization or biofilm formation [25, 26], these membrane proteins were further evaluated for their role in host cell signaling in vitro.

## Results

### Serologic cross-reactivity

No cross reaction was observed between the different rabbit polyclonal antibodies and the three recombinant proteins (P40, MAG_1560, MAG_6130) when tested via immunoassays at different concentrations (Additional file 1). Each of the three polyclonal antisera showed specific binding only to its corresponding recombinant protein and did not react with the other two proteins as depicted in Additional file 1.

### P40, MAG_1560 and MAG_6130 bind HeLa and mammary stromal cells (MSC)

#### Immunoassays with cellular fractions of MSC and HeLa cells

**Fig. 1:**
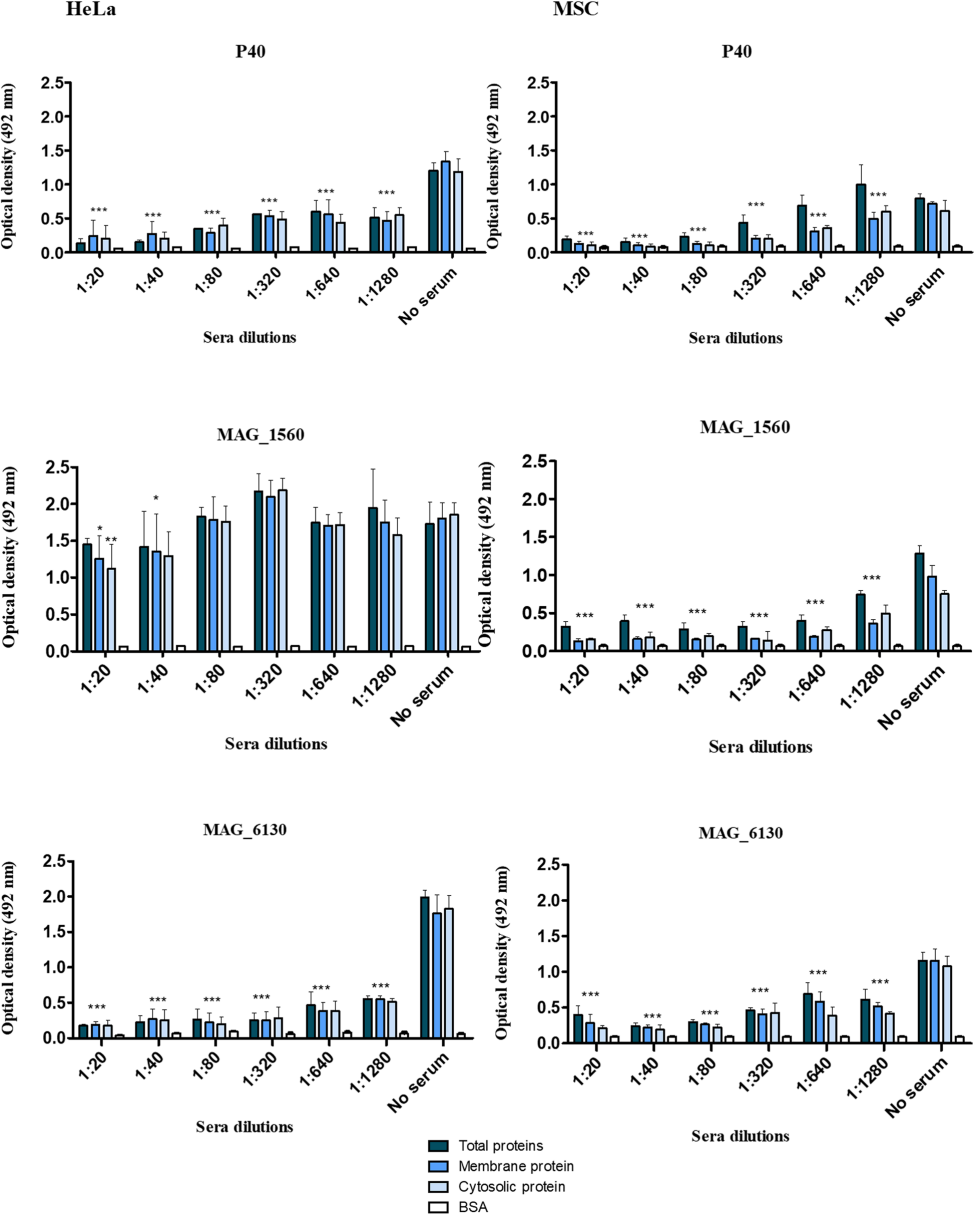
Adhesion and adhesion inhibition immunoassays of recombinant proteins P40, MAG_1560 and MAG_6130 to HeLa (left panels) and MSC (right panels) proteins. The panels show the corresponding adhesion inhibition using antisera dilutions ranging from 1:20 to 1: 1280 for each recombinant protein. BSA was used as a negative control. (*) *p* < 0.05, (**) *p* < 0.01, (***) *p* < 0.001 represent statistically significant differences

The adhesion of recombinant proteins P40, MAG_1560 and MAG_6130 to cellular fractions of HeLa and MSC was analyzed by immunoassays (Fig. 1). Total proteins, as well as the cell membrane and cytoplasmic fractions, of HeLa and MSC were incubated separately with the recombinant proteins of *M. agalactiae* to demonstrate the potential of these proteins to adhere to the different cell fractions. The inhibitory effect of anti-P40, anti-MAG_1560 and anti-MAG_6130 sera was also evaluated in these adherence assays. In case of HeLa cells, anti-P40 and anti- MAG_6130 sera (pre-incubated with the respective proteins) had a significant inhibitory effect on the adhesion of corresponding recombinant proteins and cell fractions at all tested dilutions, while the adhesion of the MAG_1560 protein was weakly inhibited only at lower dilutions of the antiserum (Fig. 1). Significant inhibitory effects were observed on adhesion of MSC total proteins and cell fractions by pre-incubating all recombinant proteins with their respective antisera, even at low concentrations (dilution 1: 1280) (Fig. 1).

#### Adhesion assays in monolayer cell culture

**Fig. 2:**
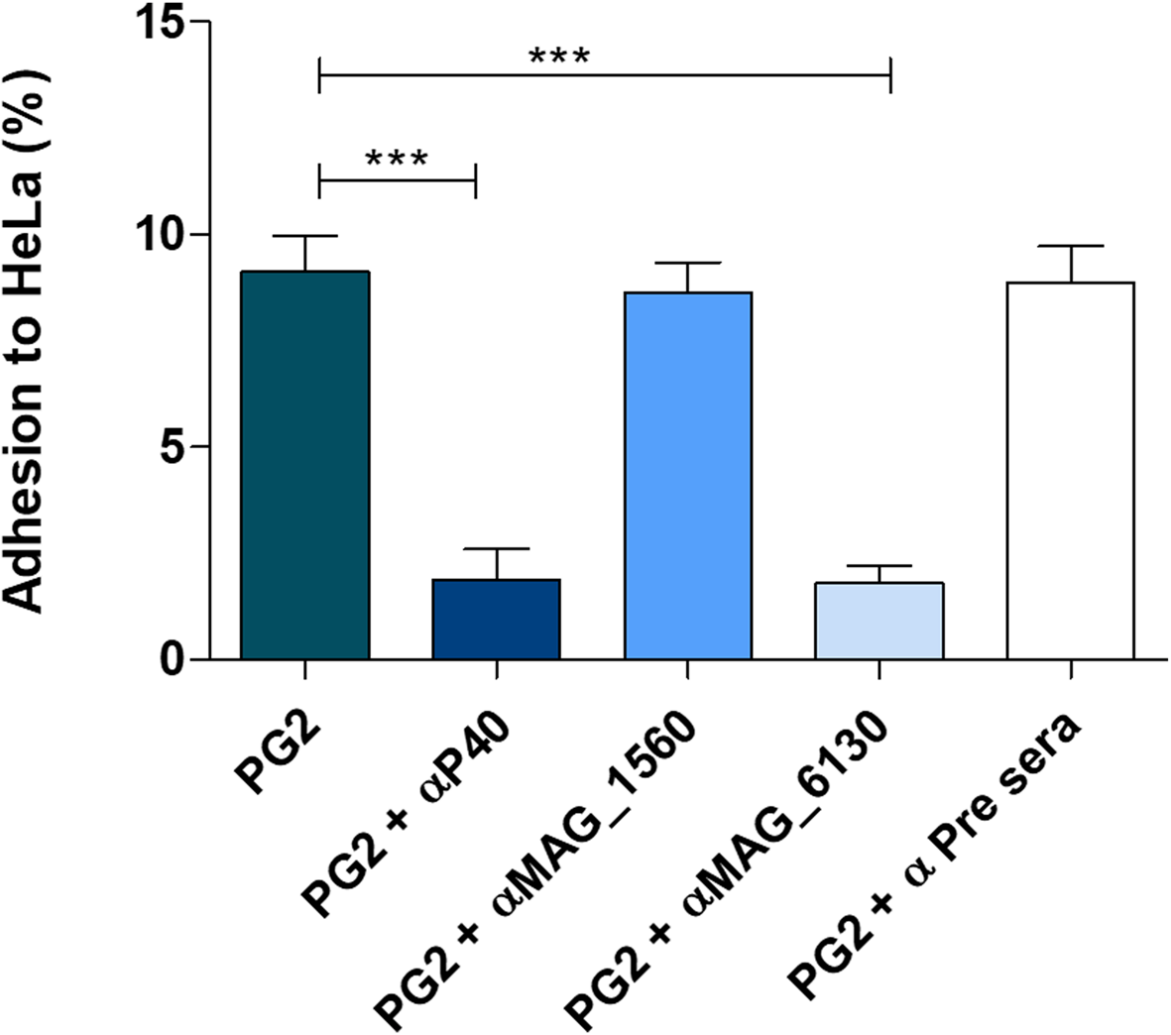
Inhibition of *M. agalactiae* adhesion to HeLa cells in monolayer cell culture. Adhesion of *M. agalactiae* strain PG2 to HeLa cells and inhibition of adhesion by pre-incubating the mycoplasma cells with the respective mono-specific antisera. Data represented as mean (± SD) of three independent experiments carried out in duplicate. Statistical analysis was performed using Student’s t test. (**) *p* < 0.01, (***) *p* < 0.001 represent statistically significant differences

Additional file 2 demonstrates the adhesion of *M. agalactiae* strains PG2 and GM139 to HeLa and sheep primary MSC cells after 4 h of infection (MOI 100). As both strains showed a similar rate of adherence for the two different eukaryotic cell cultures, adherence inhibition assays were performed with the *M. agalactiae* type strain PG2 using the standard HeLa cell line. As shown in Fig. 2, adhesion was efficiently inhibited by pre-incubating the PG2 strain with anti-P40 and anti-MAG_6130 antibodies, whereas no inhibition of adherence was observed when mycoplasma cells were pre-incubated with anti-MAG_1560 antibodies. No inhibition was observed when *M. agalactiae* was pre-incubated with pre-immune serum before adhesion assays in monolayer cell assays.

### Cell viability assay

At first the optimal incubation time and plating density were determined as recommended for the AlamarBlue™ cell viability assay described under Methodology. After standardizations, a plating density of 1 × 10^4^ cells/well for MSC and 2 × 10^3^ cells/well for HeLa was observed to produce the required reaction with the AlamarBlue™ reagent within the linear range after 48 h of incubation at 37 °C and 5% CO_2_ (Additional files 3 and 4).

**Fig. 3:**
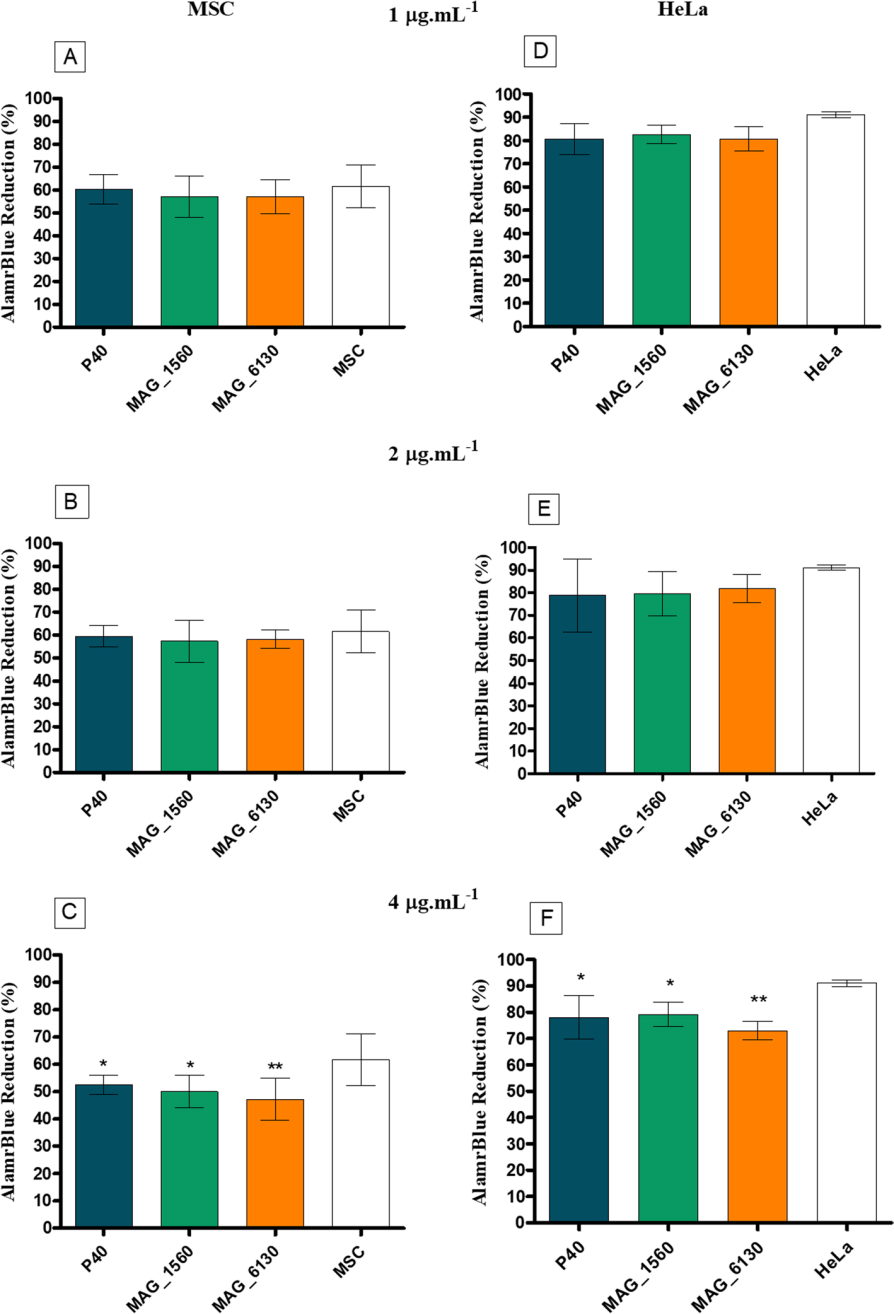
Effect of recombinant proteins of *M. agalactiae* on the viability of eukaryotic cells. The sheep MSC and HeLa cells were seeded in 96-well plates and after overnight incubation treated with (**A** and **D**) 1, (**B** and **E**) 2 and (**C** and **F**) 4 μg.mL^− 1^ of the P40, MAG_1560 or MAG_6130 proteins. After 48 h of treatment, alamarBlue™ reagent was added and its reduction was monitored spectrophotometrically at 570 nm and 600 nm. Statistical analysis was performed using Student’s t test. (*) *p* < 0.05, (**) *p* < 0.01 represent statistically significant differences

When treated with 4 μg.mL^− 1^ of P40, MAG_1560 or MAG_6130 proteins for 48 h, the MSC and HeLa (Fig. 3) cells showed a significant reduction in cell proliferation / viability (as measured by the reduction of the alamarBlue™ reagent) compared to the negative control (untreated cells). No significant reduction in cell viability was observed when incubating MSC or HeLa with the recombinant proteins at concentrations of 1 or 2 μg.mL^− 1^ (Fig. 3).

### Gene expression profile

**Fig. 4:**
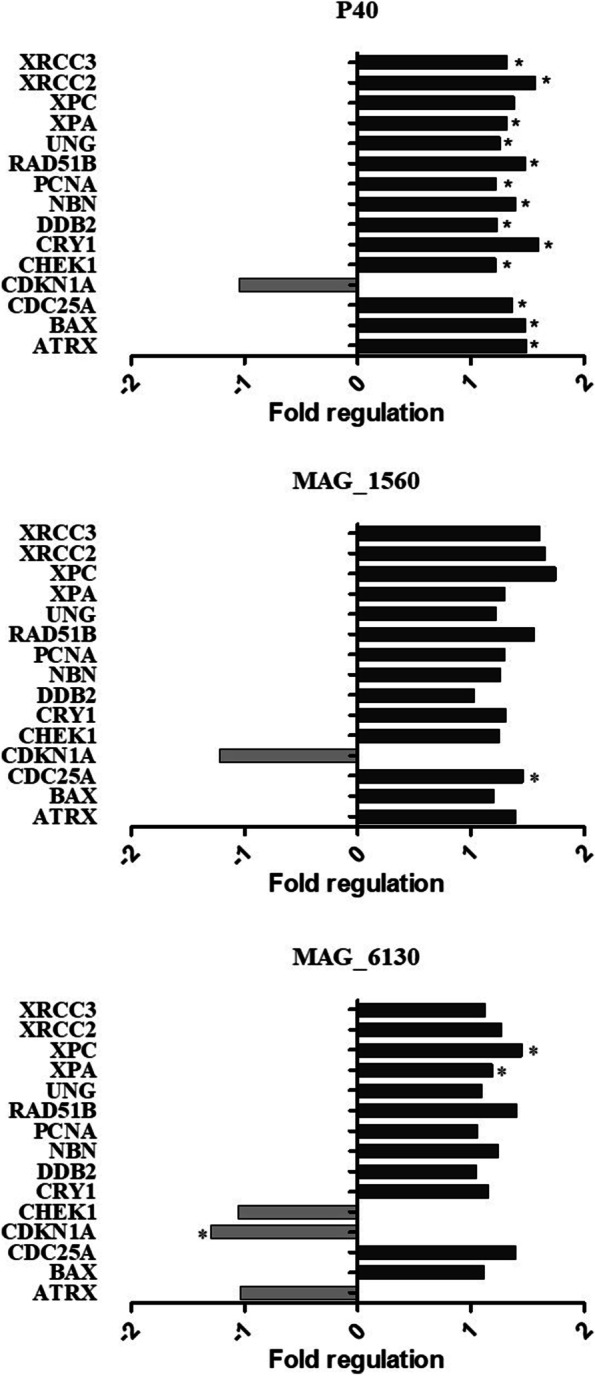
- Gene Expression profiles of cells treated with recombinant *M. agalactiae* proteins. Panels show up-regulated and down-regulated genes in cells stimulated with recombinant proteins P40, MAG_1560 and MAG_6130 for 48 h compared to the unstimulated control group. *Statistical significance (*p* < 0,05) (Student’s T-test of the replicate 2^(− Delta CT) values for each gene in the control group and treatment groups)

The expression of genes involved in DNA damage signaling pathways was evaluated in cells after 48 h incubation with proteins P40, MAG_1560 and MAG_6130 (4 μg.mL^− 1^) (Fig. 4). Among the genes analyzed, 13 genes were significantly up-regulated (ATRX, BAX, CDC25A, CHEK1, CRY1, DDB2, NBN, PCNA, RAD51B, UNG, XPA, XRCC2 and XRCC3; *p* < 0.05) in cells inoculated with P40 compared with the control unstimulated cells. In contrast, only one gene was significantly up-regulated (CDC25A; *p* < 0.05) in cells incubated with MAG_1560 and two genes (XPA and XPC; *p* < 0.05) in cells stimulated with MAG_6130 compared to the control group. As regards down-regulation, only one gene was significantly down-regulated (CDKN1A; *p* < 0.05) after stimulation with MAG 6130.

### Binding assays to host proteins

**Fig. 5:**
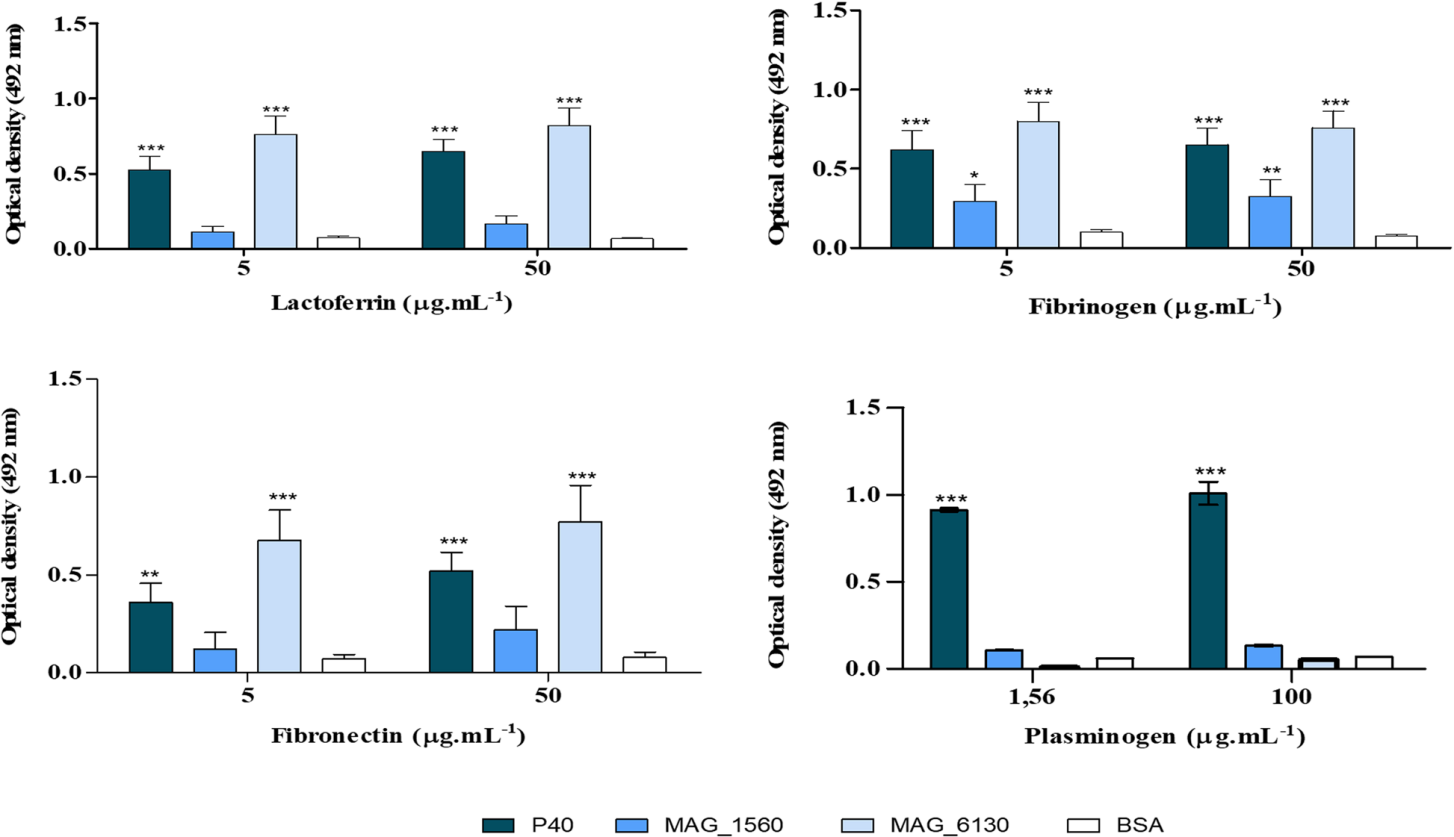
*M. agalactiae* binding to plasminogen and extracellular matrix proteins. Recombinant proteins P40, MAG_1560, MAG_6130 were applied to quantify their binding to plasminogen, fibrinogen, lactoferrin and fibronectin by indirect ELISA. Data represented with the mean (± SD). Statistical analysis was performed using Two way anova with Bonferroni post test. (***) *p* < 0.001, (**) *p* < 0.01, (*) *p* < 0.05

The graph in Fig. 5 demonstrates the binding of the P40 protein to plasminogen even at low concentration (< 2 μg.mL^− 1^ of plasminogen). On the other hand, MAG_1560 and MAG_6130 proteins do not demonstrate sufficient binding to plasminogen even at high concentrations (100 μg.mL^− 1^). However, as illustrated in Fig. 5, P40 and MAG_6130 exhibit significant binding to fibrinogen, fibronectin and lactoferrin. In contrast, MAG_1560 binds only fibrinogen. No interaction was observed between proteins and BSA controls under these conditions.

## Discussion

Mycoplasmas have several membrane proteins associated with lipids (LPPs). Unlike bacteria with cell walls, which have a lower number of these molecules, two thirds of the mycoplasma membrane mass corresponds to LPPs [4, 6, 27]. These molecules exposed to the bacterial surface may have the same functions as periplasmic proteins in Gram-negative bacteria [1] and are known to mediate adhesion [24], invasion [22], immunomodulation [5] and/or immune evasion [28], playing important roles in mycoplasma pathogenicity [21].

Cytadherence in mycoplasmas is essential for colonization and infection and considered a major pathogenicity factor. These bacteria have incomplete metabolic pathways and adhere to host cells to obtain nutrients [29]. The best characterized adhesins in mycoplasmas are those of the human mycoplasmas, *M. pneumoniae* and *M. genitalium*, and the bird pathogen *M. gallisepticum*. These microorganisms have an “adhesion organelle” which consists of a terminal structure with a central filament, formed by several adhesins [30]. Other mycoplasmas do not have a specific structure for adhesion but exhibit cytadherence capabilities mediated by other proteins, including “moonlighting” proteins, such as pyruvate dehydrogenase of *M. gallisepticum* [31]*,* fructose-1,6-bisphosphate aldolase of *M. bovis* [32]*,* elongation factor Tu of *M. pneumoniae* and *M. hyopneumoniae* [33]*,* GroEL and DnaK of *M. pneumoniae* [34]*,* P146 [35]*,* Mhp107 [36] and P116 [37] of *M. hyopneumoniae.* For *M. agalactiae,* P40 [17], pyruvate dehydrogenase [20] and Vpma lipoproteins [22] have been described as adhesins and / or invasins. In addition, it has also been demonstrated in mycoplasmas that adhesins can recognize more than one target, and one target can also bind to more than one adhesin [38].

In this study, HeLa and MSC (mammary stromal cells) cells were used in the adhesion assays. In previous studies, *M. agalactiae* PG2 strain has shown similar efficient binding to both cells [22, 39]. Current adhesion assays demonstrated that *M. agalactiae* strains PG2 and GM139 adhere to eukaryotic cells in a similar way, as shown here (Additional file 2) for both HeLa and MSC, although the adhesion rate observed in this study (between 10 and 15%) was lower than the adherence rate observed in previous studies using the same protocol and cells (approximately 33% for HeLa and 45% for MSC) [22, 39]. The difference in the adherence rate found in this study and the adherence reported in previous studies is explained solely by the experimental variation since this assay was performed in replicates and under the same conditions reported in the literature. Lower adherence rates were observed using MOI less than 100 (Additional file 5). Evaluation of the specific ability of anti-P40, anti-MAG_1560 and anti-MAG_6130 antibodies to inhibit the binding of *M. agalactiae* PG2 demonstrated that the hypothetical protein MAG_6130 has an adhesion capacity similar to P40 (Fig. 2), which is a well-known cytadhesin of *M. agalactiae* [17]. Moreover, same results were obtained using two different evaluation methods, namely inhibition of adhesion to cellular fractions in immunoassays (Fig. 1) and to monolayers of HeLa and MSC cultures (Fig. 2 and Additional file 2). On the other hand, the adhesion of MAG_1560 was inhibited only in adhesion assays using cell fractions (weakly inhibited) and not in assays employing HeLa cell monolayers (Figs. 1 and 2). Perhaps in the latter case, expression of MAG_1560 is regulated by environmental factors or there is interference between individual adhesins. Bacteria expressing multiple adhesins are known to vary their adhesion profile by controlled expression of individual adhesins at different stages of infection [40]. A possible hypothesis could be that the Hela cells do not have receptors that allow adhesion of MAG_1560, and the binding demonstrated in Fig. 1 is rather nonspecific. Though unexpected, the recombinant proteins bind to all eukaryotic cell fractions (Fig. 1) and, as already mentioned, this binding is significantly inhibited by specific hyperimmune serum (except MAG_1560 in HeLa) and not by pre-immune serum. Moreover, some other mycoplasma proteins have also been shown to bind to both cellular and cytosolic protein fractions. For instance, the recombinant NOX protein of *Mycoplasma bovis*, a close phylogenetic relative of *M. agalactiae,* was shown to bind to both the membrane proteins and cytosolic proteins of eukaryotic host cells [41].

Despite several studies of mycoplasma adhesion to host cells, little is known about the involved cell receptors and their interactions. It has been shown that extracellular β-actin [42], cyclophilin A [43], sialic acid [44, 45] and sialylated glycoconjugates [46] act as mycoplasma receptors on different cells. Future in-depth studies should be carried out to identify the receptors involved in *M. agalactiae* adhesion to eukaryotic cells, as well as receptor domains and adhesion at different stages of cell maturation.

Interactions between host proteins and mycoplasma proteins have been described in few cases [32, 34, 41, 47–50]. In this study, binding assays to host proteins demonstrated that, mainly, P40 and MAG_6130 play a role in *M. agalactiae*’s binding to these molecules (Fig. 5). P40 binds to plasminogen, but it has not been evaluated whether P40 is also able to activate plasminogen to plasmin and whether ionic interactions and the amino acid lysine interfere in this interaction. Binding and/or activation of plasminogen by mycoplasmas/recombinant proteins has been demonstrated by rMsEno (*M. synoviae)* [48], Pdh (*M. pneumoniae)* [51], PdhA and PdhB (*M. gallisepticum)* [31], Enolase (*M. hyopneumoniae*) [38], *M. fermentans* [52].

Plasminogen is immobilized on the surface of some bacteria by the presence of receptors, which allow its conversion to plasmin and assist bacterial migration through the tissue barrier [34, 49]. Additionally, plasminogen bound to the pathogen’s surface can also contribute to degradation of C3b and C5, thereby inhibiting the activation of the three pathways of the complement system [53, 54]. Thus, in addition to P40 being a cytadhesin it might as well be involved in evading the host immune response via plasminogen binding and act as a moonlighting protein. Although MAG_6130 protein does not bind to plasminogen, it can interact with molecules of the host, namely fibronectin, lactoferrin and fibrinogen, and contribute to the adhesion of *M. agalactiae* to host cells (Fig. 5). Interactions between bacterial proteins and fibronectin and fibrinogen facilitate the attachment of the microorganisms to the surface of the host cell via integrin, contributing to adhesion, invasion and formation of bacterial biofilm [26, 55]. Furthermore, binding of P40 and MAG 6130 to lactoferrin could also assist *M. agalactiae* in the acquisition of iron for growth and protection against cationic antimicrobial peptides as described for other bacterial pathogens [56]. Overall, the hypothetical protein MAG_6130 is not only involved in *M. agalactiae’s* adhesion to host cells like the cytadhesin P40, it might as well play other important roles in the pathogen’s survival and immune evasion capabilities inside the host, and act as a “moonlighting” protein.

Adhesion of mycoplasmas to host cells can lead to cell damage (Rotten, 2003). *M. agalactiae* has been earlier demonstrated to induce cytopathic effects in infected host cells [57]. This study shows that P40, MAG_1560 and MAG_6130 are capable of altering the cell viability / proliferation of eukaryotic host cells (Fig. 3), thereby contributing to the process of cell destruction in host cells infected with *M. agalactiae*. A similar effect of membrane proteins on cell destruction has been reported in other mycoplasmas [58]. In *M. pneumoniae* the absence of phosphorylation in HMW1, HMW3, the major adhesin P1, and the surface protein MPN474 alters the function of the terminal organelle resulting in decreased adherence and loss of cytotoxicity [59]. Further studies are needed to assess whether these membrane proteins are involved in cell destruction by inducing pro-inflammatory cytokines (IL-1 and IL-6), NO and ROS.

Apoptotic events have been demonstrated to occur in several pathogenic animal mycoplasma species [57, 60–65] and human mycoplasmas [66] by the activation of caspases, MAPK, ROS, and the ERK signaling pathway [10, 67]. More specifically, it has been shown that certain proteins, such as MbovNase nuclease [68]*,* P48 [69] and MbovP280 [70] of *M. bovis;* Mhp597 and P68 of *M. hyopneumoniae* [71, 72] and GroEL, of *M. gallisepticum* [73] trigger pro-apoptotic genes by the activation of MAPK, BAK, and caspases or by unknown pathways. In this study, we demonstrate a similar up-regulation profile of genes in cells stimulated by membrane proteins of *M. agalactiae*, mainly by P40. For instance, an increased expression of *ATRX*, which acts on the remodeling of chromatin and is related to the MAPK cascade [74] was observed. Also, the expression of *Bax*, a known activator of caspase 9 and caspase 3 that allows release of cytochrome c and other molecules through channels in the mitochondrial membrane was enhanced [75]. Altogether, these data point towards the occurrence of pro-apoptotic events. Additionally, an increase in the expression of molecules that act in response to DNA damage was observed. Initially RAD51 is recruited in a manner dependent on ATM and NBN, the latter guides MRE11A and RAD50 to the DNA damage site, where it interacts with the ATM protein [76, 77]. RAD51 in complex with XRCC3, promotes the activation of Chek2. ATR is also activated and phosphorylates Chek1 resulting in the interruption of the cell cycle. DDB2 also downregulates p21 by proteolysis, allowing cell death [78]. Overall, due to the severity of the damage, the cell undergoes premature apoptosis [79, 80]. Moreover, plasminogen binding has also been reported to be associated with an increased rate of apoptosis [81].

## Conclusion

Only a few *M. agalactiae* proteins have been functionally characterized to play important roles in its pathogenicity, including P40 [17], P30 [16], PdhB [20], Vpmas [22] and MAG_5040 [13]. In this study, hypothetical protein corresponding to MAG_6130 has not only been assigned novel adhesion functions but together with P40 it is demonstrated for the first time to bind lactoferrin and ECM proteins. All these characteristics could have far-reaching effects on the pathogenicity as also seen for PavB of *Streptococcus pneumoniae*, which is also an adhesin, similarly interacts with plasminogen and fibronectin, and its mutants were demonstrated to be attenuated and out-competed by wild type strain in a mice co-infection study [82]. Furthermore, P40 binds plasminogen and was shown to induce DNA damage. Overall, these multifunctional proteins may contribute to colonization, immune evasion, and establishment of the *M. agalactiae* infection, and are anticipated to serve as important serodiagnostic and vaccine candidates.

## Methodology

### Bacterial strains, cell lines and culture conditions

*Mycoplasma agalactiae* strain GM139 [83] and type strain PG2 were grown at 37 °C in SP4 medium supplemented with penicillin and phenol red as described earlier [84]. For cell infections, HeLa-229 cells (CCL-2.1, ATCC, USA) and sheep primary mammary stromal cells (MSCs) (MSC cells were obtained from an adult lactating sheep and characterized via immunohistochemistry in a previous study [39] and were stored in liquid nitrogen until the moment of use) were cultured as reported previously [39]. Briefly, HeLa cells were cultured in MEM medium containing 10% heat-inactivated fetal bovine serum and MSC cells were cultured in DMEM high glucose (89%) medium containing 1% L-glutamine and 10% heat-inactivated fetal bovine serum. For adhesion assays, 5 × 10^4^ cells/well were plated on 24-well plates (CELLSTAR®, Greiner Bio-One GmbH, Germany) 48 h before infection. For cell viability tests, 1 × 10^4^ cells/well for MSC and 2 × 10^3^ cells/well for HeLa were plated in 96-well plates 24 h before inoculation. HeLa and MSC cells were used at passage 25 and passage 6, respectively. The cell cultures were periodically tested for mycoplasma contamination by culture and/ or PCR [85].

### Expression and purification of recombinant proteins

Recombinant proteins [P40, MAG_1560 (MAG_RS00795), MAG_6130 (MAG_RS03125)] were expressed in *Escherichia coli* and purified on affinity columns as described by Barbosa et al. (2020) [23]. Briefly, *E. coli* BL21 Star™ (DE3) One Shot containing the expression vector (pET28a) was cultured in medium containing kanamycin and IPTG, and the proteins were purified using nickel chelating resin (HisTrap™ HP, GE Healthcare Bio-Sciences Corp., USA). Proteins were assessed by 12% SDS-PAGE stained with Coomassie Blue and Western blots using the primary antibody against the 6x-His Epitope Tag (Invitrogen™) (Additional file 6) and subjected to membrane dialysis.

### Mono-specific polyclonal antibodies

Polyclonal antibodies were produced in New Zealand rabbits as ethically approved (FMUSP – 944/2017; ICB – 123/2016 /CEUA) and described earlier [23]. All methods were performed in accordance with the relevant guidelines and regulations. Briefly, rabbits were first immunized with 500 μg of recombinant protein emulsified in complete Freund’s adjuvant (Sigma-Aldrich®) (v/v). Subsequently, two additional immunizations were performed at two-week intervals. On the 42nd day of immunization, the animals were submitted to cardiac puncture exsanguination. The purification of antisera using G protein columns and the titer were realized previously by ELISA [23].

Cross-reactivity between polyclonal antibodies and the P40, MAG_1560, MAG_6130 proteins was assessed by immunoassays. The latter were performed on polystyrene plates (Nunc™, Thermo Scientific™) coated with 500, 1000 and 2000 ng.mL^− 1^ of each recombinant protein separately after dilution in carbonate-bicarbonate buffer pH 9.6 (100 μL/well) for 16 h at 4 °C in humid chamber. The plates were washed with TBS - Tween 20 (TBST) (0.05%) and non-specific binding sites were blocked for one hour at 37 °C with 5% skimmed milk in TBST (200 μL/well). The plates were rewashed and the mono-specific polyclonal antibodies were added (100 μL/well) at different dilutions (0.5 μg.mL^− 1^, 0.25 μg.mL^− 1^,0.125 μg.mL^− 1^). Then the microplates were incubated at room temperature for 1.5 h. Subsequently, the microplates were washed again and the secondary antibody conjugated with peroxidase (Goat anti-Rabbit IgG, HRP conjugate - Invitrogen™) was added at a dilution of 1:5000 in TBST containing 5% skimmed milk (100 μL/well). After incubation at room temperature for 1.5 h, the plates were washed again. The reactions were developed using the chromogenic substrate OPD (o-Phenylenediamine Dihydrochloride, Thermo Scientific™) with the addition of hydrogen peroxide for 10 min. The reaction was stopped with 50 μL of 1 N sulphuric acid before optical density (O.D.) measurements were recorded on a microplate reader at 492 nm.

### In vitro adhesion assays

#### Immunoassays

To test the quantitative binding of recombinant proteins to HeLa and MSC an immunoassay was used. HeLa and MSC were used since the ability of *M. agalactiae* to adhere to both these host cells is previously known [22, 39]. Initially, 96-well plates were coated at 4 °C overnight with proteins (10 μg/well): total cell proteins, cell membrane or the cytosolic fractions of HeLa or MSC in bicarbonate-carbonate sodium buffer (pH 9.6). The protein fractions of eukaryotic cells i.e. HeLa and MSC, were obtained after extraction with 1% Triton TX-114 as previously described [86, 87]. For the adhesion test, the wells were blocked with 5% milk before adding 1000 or 2000 ng.mL^− 1^ of recombinant proteins diluted in TBST (100 μL/well). The reaction was incubated at 37 °C for 1.5 h and the wells washed thrice with TBST followed by incubation with the respective anti-recombinant protein antibody at room temperature for 1.5 h, anti-P40 (5 μg.mL^− 1^), anti-MAG_1560 (0.1 μg.mL^− 1^), and anti-MAG_6130 (1 μg.mL^− 1^) (100 μL/well). After washing, anti-rabbit IgG-HRP antibody (1: 5000; 100 μL/well) was added and the plate again incubated at room temperature for 1.5 h before recording the reaction as described above. BSA was used as a negative control.

For the adhesion inhibition assays, each antiserum against the specific recombinant protein (1 mg.mL^− 1^) was serially diluted from 1/20 to 1/1280. Each of these dilutions (100 μL) were pre-incubated with 1000-2000 ng.mL^− 1^ of recombinant protein in 100 μl TBST at 37 °C for 1 h. Subsequently, each mixture was added to the wells previously coated with the eukaryotic cells’ protein fractions. The reaction and detection proceeded as described above [34, 41].

#### Monolayer cell cultures

*M. agalactiae* strains PG2 and GM139 were incubated with HeLa and MSC (at a MOI of 100, as previously described by Hegde et al., 2015a, 2018 [22, 39]) for 4 h at 37 °C and 5% CO_2_ to assess their cell adhesion capacity. Non-adhered mycoplasmas were removed by three washes with PBS and serial dilutions of the cell suspension plated on SP4 agar after trypsinization. As controls, mycoplasma suspensions were incubated in the absence of eukaryotic cells in parallel wells to quantify the CFU after 4 h of incubation. Adherence was calculated using the ratio of the CFU.mL^− 1^ of the adhered mycoplasmas to the CFU.mL^− 1^ of total mycoplasmas in the given time [22, 39].

For the adhesion inhibition assays, *M. agalactiae* was separately pre-incubated at 37 °C for 1 h with each of the three antisera (10:1, v/v) against the specific recombinant proteins. The mycoplasma-antibody suspension was then added to the eukaryotic cells and further incubated at 37 °C, 5% CO_2_ for 4 h. The percentage adherence was calculated as described above [22].

### Cell viability assays

Initially the optimal incubation time and plating density was determined. For that, 2.5 × 10,^3^ 5 × 10,^3^ 1 × 10^4^ and 2 × 10^4^ cells/ well (MSC) or 5 × 10,^2^ 1 × 10,^3^ 2 × 10,^3^ 2.5 × 10^3^ and 5 × 10^3^ HeLa cells/ well were incubated overnight. After washing the cells with PBS, 90 μL of media followed by 10 μL of alamarBlue™ HS Cell Viability Reagent Invitrogen™ was added to each well. The plates were incubated at 37 °C, 5% CO_2_ for 24 h, 48 h and 72 h. The absorbance of the reaction was measured at a wavelength of 570 nm and 600 nm at each hour for 8 h, 10 h and 24 h after incubation with the reagent. The percentage reduction of alamarBlue™ reagent using absorbance readings was calculated following manufacturer’s instructions.

For the cell viability assay, 1 × 10^4^ MSC cells/ well or 2 × 10^3^ HeLa cells/ well were plated in 96 well plates. After overnight incubation at 37 °C and 5% CO_2_, the cells were washed with PBS and incubated with recombinant proteins P40, MAG_1560 or MAG_6130 (1, 2 and 4 μg.mL^− 1^; 100 μL/well) for 48 h under the same conditions. Prior to use in cell stimulation, the recombinant proteins were filtered through 0.22 μm filters and preincubated for 2 h with polymyxin B (lipopolysaccharide-neutralizing agent) at 1000 U.mL^− 1^ [10]. Subsequently, the alarmarBlue™ reagent was added to each well and readings recorded after every hour, for 4-6 h using a microplate reader to calculate the percentage of reduction of the alarmarBlue™ reagent as described above.

### Gene expression analysis

Gene expression of the DNA damage-signaling pathway was evaluated by qPCR array methodology. The mRNA was extracted using RNAeasy mini Kit (Qiagen-SABioscience) following the protocol provided by the manufacturer. The cDNA was obtained by means of a retro-transcription (RT) from the mRNA, using the SuperScript™ IV Reverse Transcriptase kit with addition of oligonucleotides complementary to the poly-A tail of the mRNA, (Oligo dT) and inhibitor of RNAse. The obtained cDNA was subjected to analysis with the use of RT^2^ Profiler™ qPCR Array Human DNA Damage Signaling Pathway kit (Qiagen-SABioscience) for the expression of 84 genes involved in the host response to DNA damage. All procedures, data analysis and statistical analysis were performed according to the manufacturer’s instructions and software Qiagen-SABioscience (https://dataanalysis.qiagen.com/pcr/arrayanalysis.php). The data are presented in fold change values for each gene relative to expression in the control group (basal expression) and the stimulated group.

### Binding assays to host proteins

The plasminogen binding assay was performed in 96-well plates covered with recombinant proteins P40, MAG_1560 or MAG_6130 (500 ng/100 μL/ well) diluted in bicarbonate carbonate buffer pH 9.6 for 16 h at 4 °C, in a humid chamber, followed by blocking with 5% milk in TBST (200 μL/well) for 2 h at 37 °C. After five washes with TBST, the wells were incubated with different concentrations of bovine plasminogen (1.562 and 100.0 μg.mL^− 1^; 100 μL/well) (Sigma-Aldrich®) in PBS pH 7.4 at 37 °C for 1.5 h. Binding to plasminogen was detected by the addition of 100 μL/well of 1: 2000 diluted rabbit anti-plasminogen IgG (Abcam). Wells incubated with BSA served as negative control for plasminogen binding. The reactions were quantified as described earlier [34, 41]. Bovine plasminogen is similar to plasminogen from goats and sheep (coverage > 92% and identity > 88%).

For the protein binding assays, extracellular matrix (ECM) proteins (fibronectin and fibrinogen) and lactoferrin (Sigma-Aldrich®) (5 and 50.0 μg.mL^− 1^) were individually diluted in bicarbonate carbonate buffer pH 9.6 and added 100 μL/ well in 96-well plates for 16 h at 4 °C, in a humid chamber, followed by blocking with 5% milk in TBST (200 μL/ well) for 2 h at 37 °C. After five washes with TBST, the wells were incubated with recombinant proteins P40, MAG_1560 and MAG_6130 (500 ng/100 μL/ well) in PBS pH 7.4 at 37 °C for 1.5 h. The plates were re-washed and the mono-specific polyclonal antibodies were added at 1:1600 dilution in TBST and incubated for 1 h at 37 °C. Subsequently, the microplates were washed again and the binding was detected by the addition of diluted anti-Rabbit IgG HRP conjugate 1: 10000 (100 μL/ well) (Sigma-Aldrich®). Wells incubated with BSA were used as negative controls for binding [88]. The reactions were quantified as described above.

### Statistical analysis

Statistical analysis was performed using the GraphPad-Prism 6.0 program (GraphPad Software, USA). To evaluate the antigen-antibody cross-reaction and adhesion to plasminogen, lactoferrin and ECM proteins, the non-parametric Two way ANOVA test with Bonferroni post test was performed. To evaluate the adhesion between recombinant proteins and fractions of eukaryotic cells, One way ANOVA non-parametric test with Dunnett post test was used, whereas to analyze the inhibition of *M. agalactiae* adhesion in monolayer cell cultures and cell viability, Student’s t test was performed. The statistical analyses were assessed from at least two independent experiments carried out in duplicates or triplicates. Data is expressed as mean ± standard deviation. Statistical differences were considered significant when *p* < 0.05 using a 95% confidence interval.

## Supplementary Information

**Additional file 1. **Analysis of cross reactivity of the three rabbit antisera. Cross reactivity between recombinant proteins (P40, MAG_1560 and MAG_6130) at concentrations of 500, 1000 and 2000 μg.mL^− 1^ and the corresponding rabbit polyclonal antibodies evaluated via immunoassays at concentrations of 0.5, 0.25 and 0.125 mg.mL^− 1^. Two way ANOVA test with Bonferroni post test was performed. Data expressed as mean ± standard deviation. (***) *p* < 0.001.

**Additional file 2. **Adhesion of *M. agalactiae* strains PG2 and GM139 to HeLa and sheep primary mammary stromal cells - MSCs. Adhesion rate of *M. agalactiae* after 4 h of infection to HeLa and MSC cells (MOI 100). Data represent the mean (± SD) of three independent experiments carried out in duplicate. Statistical analysis was performed using One way Anova with Dunnett post test.

**Additional file 3.** Percentage reduction of alamarBlue™ reagent in MSC at different cell numbers and incubation times. Four different amounts of cells per well were plated and incubated at (A) 24 h, (B) 48 h and (C) 72 h at 37 °C, 5% CO_2_. The alamarBlue™ reagent (10 μL/well) was added and the readings taken at 570 nm and 600 nm to determine the optimal incubation time and plating density.

**Additional file 4.** Percentage reduction of alamarBlue™ reagent in HeLa at different cell numbers and incubation times. Five different amounts of cells per well were plated and incubated at (A) 24 h and (B) 48 h at 37 °C, 5% CO_2_. The alamarBlue™ reagent (10 μL/well) was added and readings taken at 570 nm and 600 nm to determine the optimal incubation time and plating density.

**Additional file 5. **Adhesion of *M. agalactiae* type strain PG2 to HeLa. Adhesion rate of *M. agalactiae* after 4 h of incubation with HeLa cells using different MOI. Data represent the mean (± SD) of three independent experiments carried out in duplicate.

**Additional file 6. **Purity profile of the three recombinant proteins of *Mycoplasma agalactiae.* A) 12%-polyacrylamide gel electrophoresis stained with Coomassie Blue, MW: Molecular weight Novex® Sharp Unstained Protein Standard (Invitrogen™, USA). B) Western blot performed with anti-histidine antibody (6x-His Epitope Tag, Invitrogen™, USA), MW: Molecular weight Novex® Sharp Pre-stained Protein Standard (Invitrogen™, USA). Lane 1: P40 (42 KDa); Lane 2: MAG_1560 (32 KDa); Lane 3: MAG_6130 (24 KDa).

## Data Availability

The datasets used and/or analysed during the current study are available from the corresponding author upon reasonable request.

## Abbreviations

ECM: Extracellular matrix
IPTG: Isopropyl-β-d-thiogalactopyranoside
LPP: Lipid-associated membrane proteins
MOI: Multiplicity of infection
MSC: Sheep primary mammary stromal cells
O.D.: Optical density
OPD: o-Phenylenediamine Dihydrochloride
SD: Standard deviation
SDS: Sodium dodecyl sulfate
SDS-PAGE: (SDS)-polyacrylamide gel
Vpma: Variable proteins of *Mycoplasma agalactiae*
