# Supplementary figures and images for: Host cell interactions of novel antigenic membrane proteins of Mycoplasma agalactiae
Source: BMC Microbiol. 2022 Apr 8;22:93. doi: 10.1186/s12866-022-02512-2 (PMC8991494; doi:10.1186/s12866-022-02512-2)

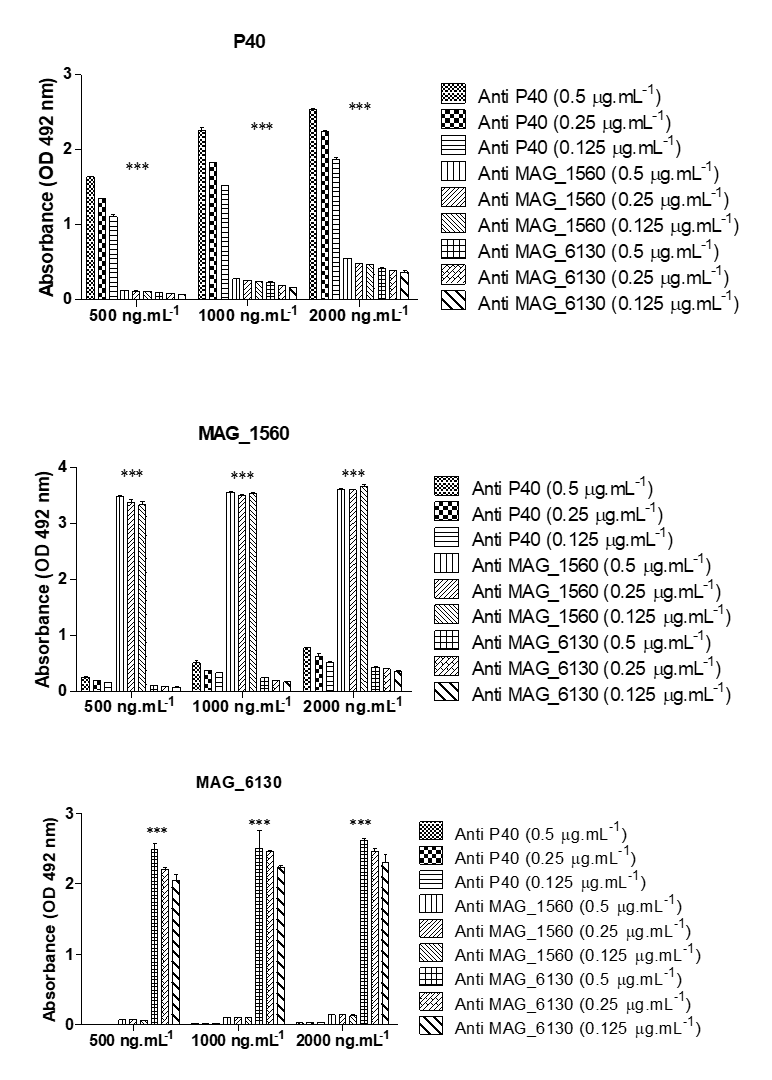

Supplement: Supplementary file 1 — Additional file 1. Analysis of cross reactivity of the three rabbit antisera. Cross reactivity between recombinant proteins (P40, MAG_1560 and MAG_6130) at concentrations of 500, 1000 and 2000 μg.mL− 1 and the corresponding rabbit polyclonal antibodies evaluated via immunoassays at concentrations of 0.5, 0.25 and 0.125 mg.mL− 1. Two way ANOVA test with Bonferroni post test was performed. Data expressed as mean ± standard deviation. (***) p < 0.001. [file 12866_2022_2512_MOESM1_ESM.docx]

**
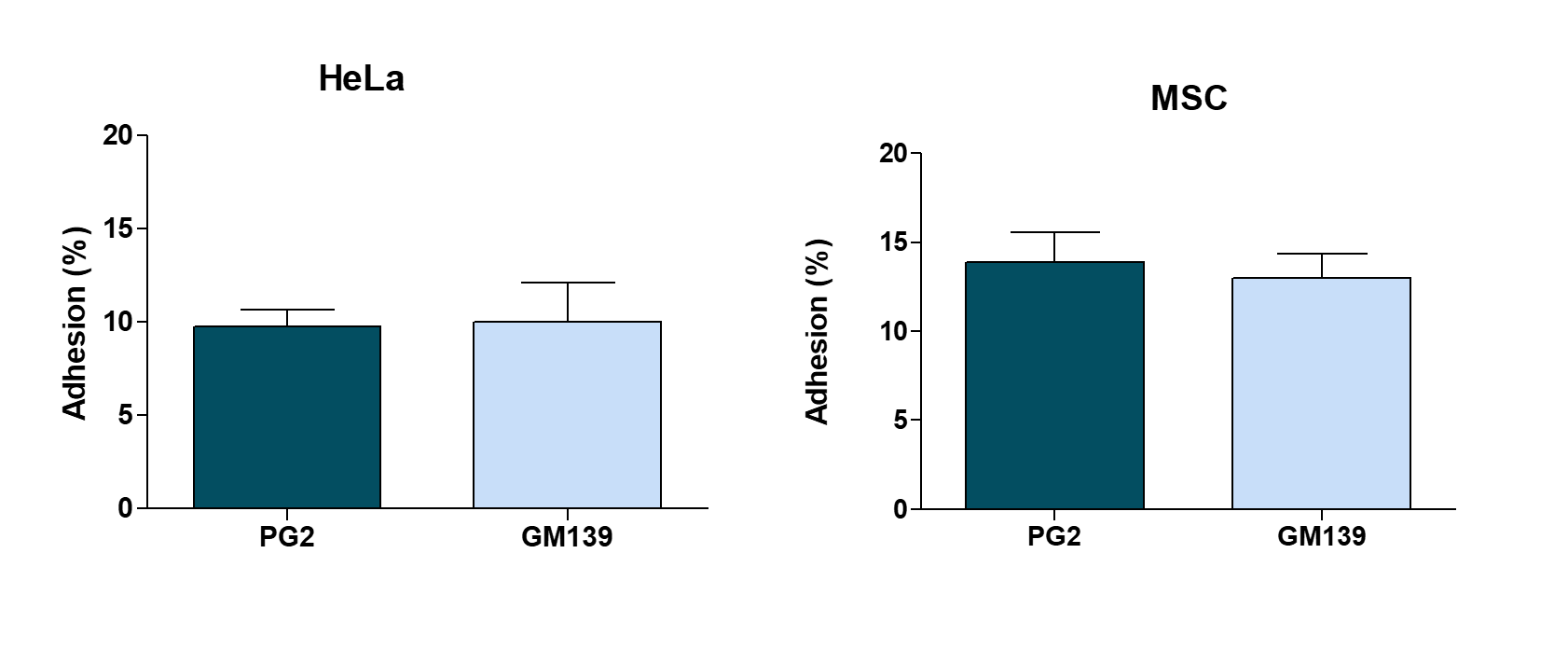
**

Supplement: Supplementary file 2 — Additional file 2. Adhesion of M. agalactiae strains PG2 and GM139 to HeLa and sheep primary mammary stromal cells - MSCs. Adhesion rate of M. agalactiae after 4 h of infection to HeLa and MSC cells (MOI 100). Data represent the mean (± SD) of three independent experiments carried out in duplicate. Statistical analysis was performed using One way Anova with Dunnett post test. [file 12866_2022_2512_MOESM2_ESM.docx]

**
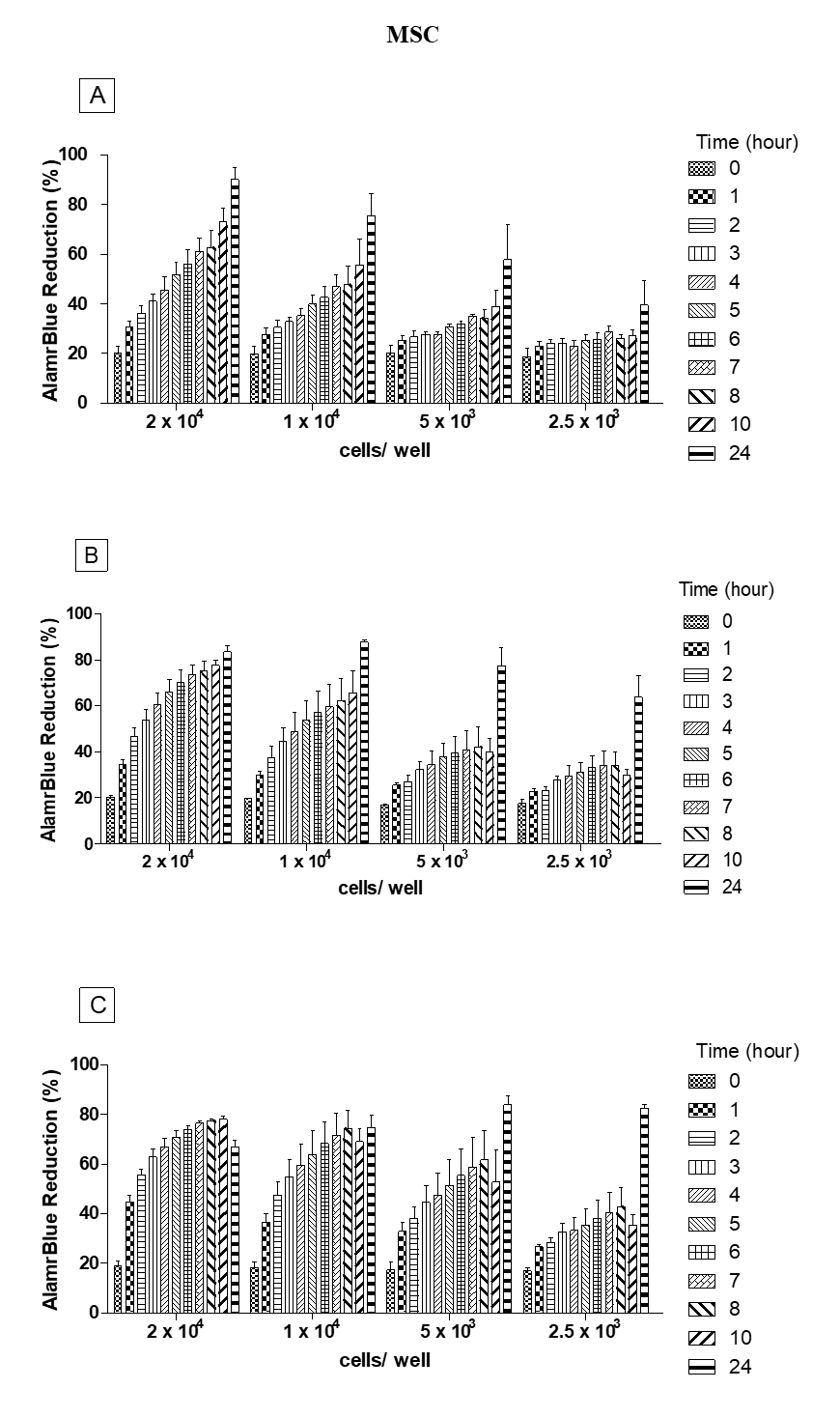
**

Supplement: Supplementary file 3 — Additional file 3. Percentage reduction of alamarBlue™ reagent in MSC at different cell numbers and incubation times. Four different amounts of cells per well were plated and incubated at (A) 24 h, (B) 48 h and (C) 72 h at 37 °C, 5% CO2. The alamarBlue™ reagent (10 μL/well) was added and the readings taken at 570 nm and 600 nm to determine the optimal incubation time and plating density. [file 12866_2022_2512_MOESM3_ESM.docx]

**
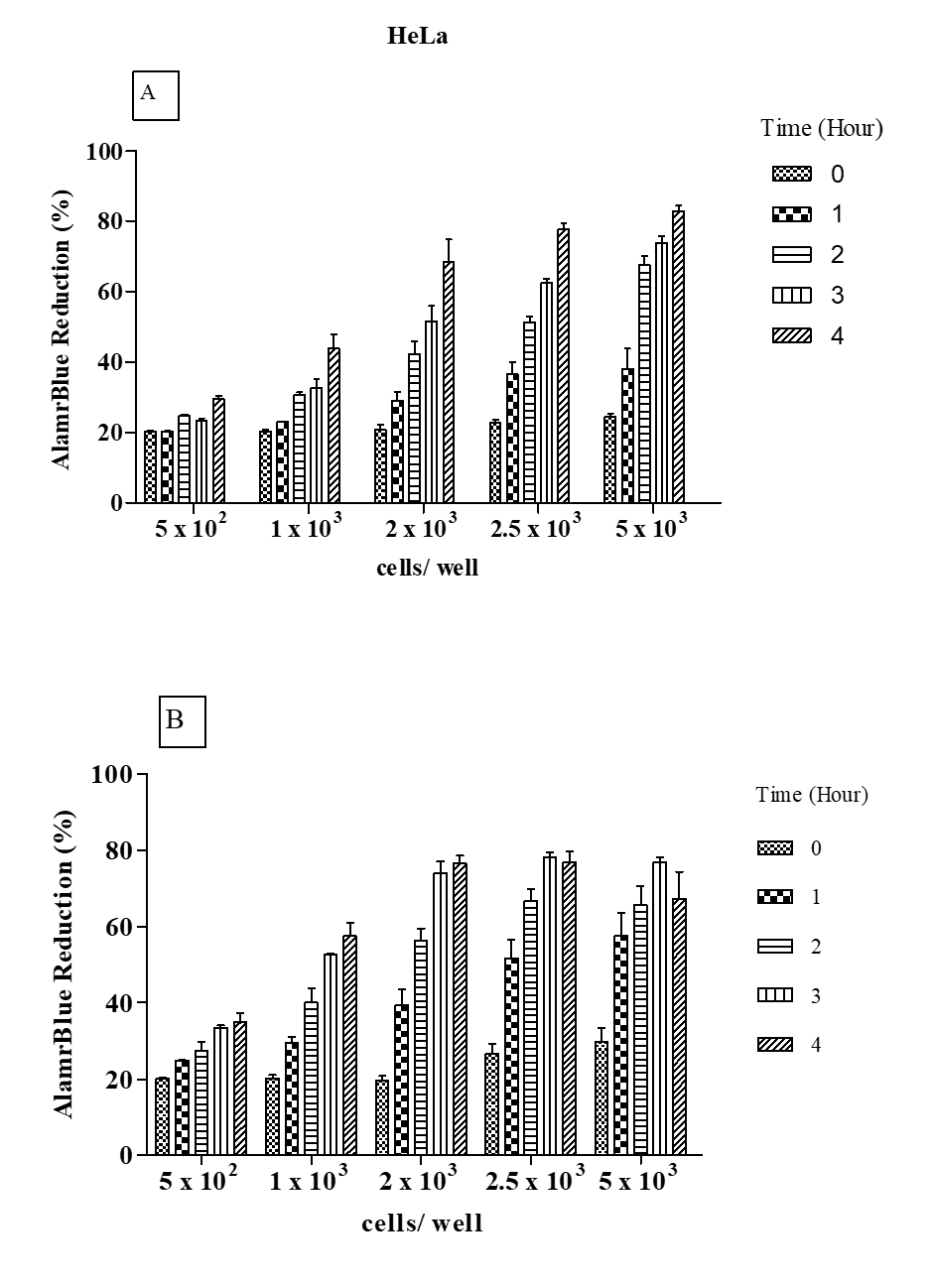
**

Supplement: Supplementary file 4 — Additional file 4. Percentage reduction of alamarBlue™ reagent in HeLa at different cell numbers and incubation times. Five different amounts of cells per well were plated and incubated at (A) 24 h and (B) 48 h at 37 °C, 5% CO2. The alamarBlue™ reagent (10 μL/well) was added and readings taken at 570 nm and 600 nm to determine the optimal incubation time and plating density. [file 12866_2022_2512_MOESM4_ESM.docx]

**
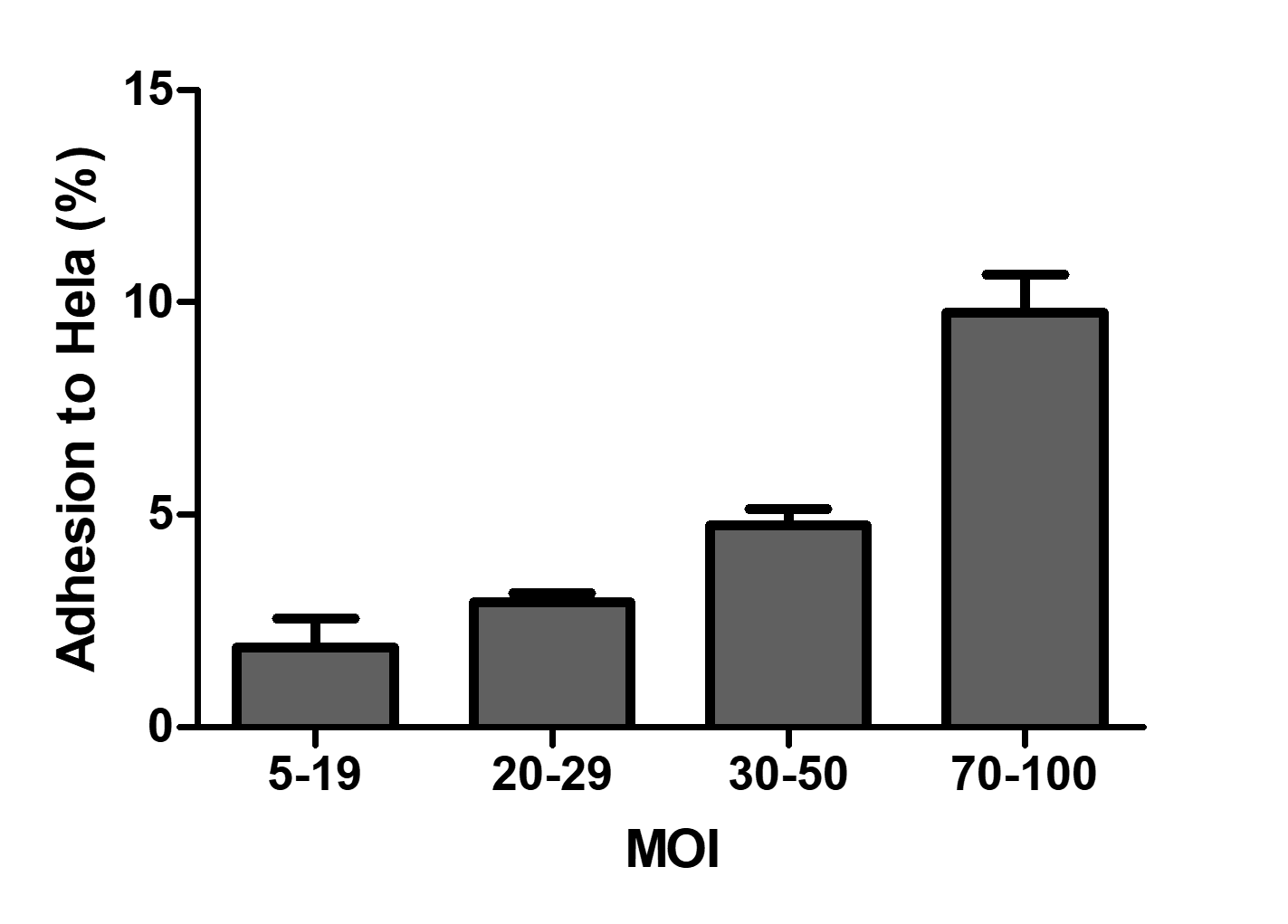
**

Supplement: Supplementary file 5 — Additional file 5. Adhesion of M. agalactiae type strain PG2 to HeLa. Adhesion rate of M. agalactiae after 4 h of incubation with HeLa cells using different MOI. Data represent the mean (± SD) of three independent experiments carried out in duplicate. [file 12866_2022_2512_MOESM5_ESM.docx]

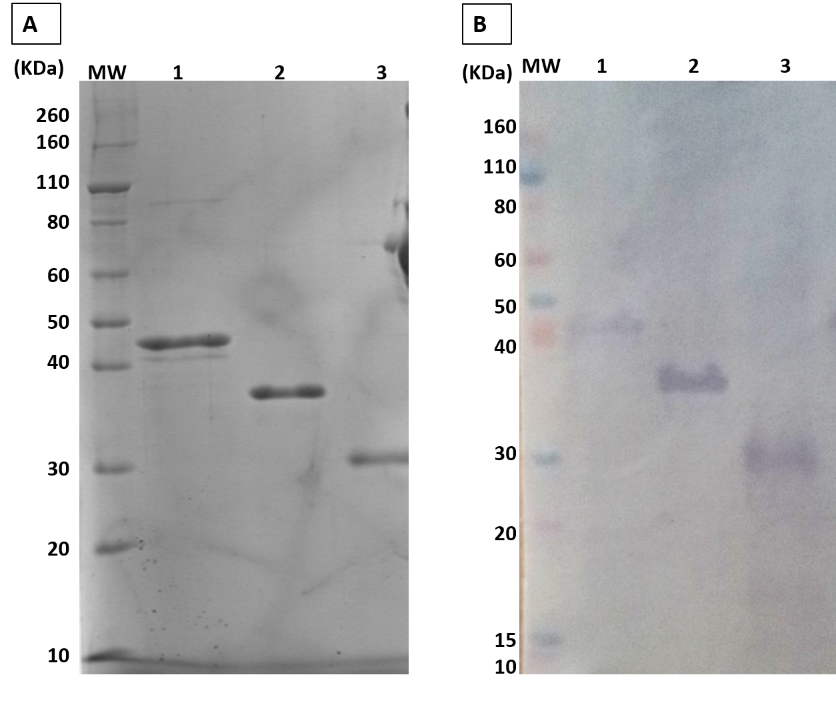

Supplement: Supplementary file 6 — Additional file 6. Purity profile of the three recombinant proteins of Mycoplasma agalactiae. A) 12%-polyacrylamide gel electrophoresis stained with Coomassie Blue, MW: Molecular weight Novex® Sharp Unstained Protein Standard (Invitrogen™, USA). B) Western blot performed with anti-histidine antibody (6x-His Epitope Tag, Invitrogen™, USA), MW: Molecular weight Novex® Sharp Pre-stained Protein Standard (Invitrogen™, USA). Lane 1: P40 (42 KDa); Lane 2: MAG_1560 (32 KDa); Lane 3: MAG_6130 (24 KDa). [file 12866_2022_2512_MOESM6_ESM.docx]
